# Wild bees (Apoidea, Anthophila) of south-west France: more than 10 years of inventories in mosaic landscapes of "Vallées et Coteaux de Gascogne" (ZA-PYGAR)

**DOI:** 10.3897/BDJ.12.e135157

**Published:** 2024-11-29

**Authors:** Mélodie Ollivier, Justine Rivers-Moore, Magalie Pichon, Emilie Andrieu, Romain Carrié, Rémi Rudelle, Jean-Pierre Sarthou, Annie Ouin

**Affiliations:** 1 UMR DYNAFOR INP-ENSAT, 31320 Auzeville-Tolosane, France UMR DYNAFOR INP-ENSAT 31320 Auzeville-Tolosane France; 2 LTSER Zone Atelier “PYRÉNÉES GARONNE”, 31320 Auzeville-Tolosane, France LTSER Zone Atelier “PYRÉNÉES GARONNE” 31320 Auzeville-Tolosane France; 3 Facultad de Agronomía y Sistemas Naturales, Pontificia Universidad Católica de Chile, Santiago, Chile Facultad de Agronomía y Sistemas Naturales, Pontificia Universidad Católica de Chile Santiago Chile; 4 UMR DYNAFOR INRAE, 31320 Auzeville-Tolosane, France UMR DYNAFOR INRAE 31320 Auzeville-Tolosane France; 5 Rudélide Expertise Muséologie, Rieupeyroux, France Rudélide Expertise Muséologie Rieupeyroux France; 6 Centre de Recherche sur la Biodiversité et l'Environnement (CRBE), Université de Toulouse, CNRS, IRD, Toulouse INP, Université Toulouse 3 – Paul Sabatier (UT3), Toulouse, France Centre de Recherche sur la Biodiversité et l'Environnement (CRBE), Université de Toulouse, CNRS, IRD, Toulouse INP, Université Toulouse 3 – Paul Sabatier (UT3) Toulouse France

**Keywords:** Hymenoptera, Apoidea, Anthophila, wild bees, south-west France, ZA-PYGAR, long-term monitoring, DNA barcoding

## Abstract

**Background:**

The reported massive decline of arthropods and particularly of pollinators such as wild bees, in terms of abundance and richness, is a threat for crop production and wild plant biodiversity conservation. This decline is mainly explained by a combination of drivers at local- and landscape-scale related to intensive farming practices. Assessing the evolution of wild bee communities in agricultural ecosystems and their response to such practices is needed to address conservation purposes.

**New information:**

We provide here data for the 24,329 wild bee specimens held in the collection of DYNAFOR Lab (UMR 1201 INRAE, INP-ENSAT, EI PURPAN), located at INP-ENSAT (Toulouse, France). All bee specimens were collected from the long term socio-ecological research site, ZA-PYGAR, located in south-west France, for more than 10 years (2010 to 2022) within the framework of different research programmes conducted by the DYNAFOR Lab. At least 270 species, representative of the six wild bee families, were identified from this area. The identified specimens are considered reliable as identifications were performed or have been verified by community-recognised experts. In addition, ongoing DNA barcoding performed on certain specimens helped clarify questionable morphological characters and provided cross-validation of species identification.

## Introduction

The worldwide decline of pollinators combined with the growing demand in pollination for crop production in quantity, but also in nutritional quality has led to an increase in research studies on pollinators and particularly on wild bees (Kremen et al. 2002, Seibold et al. 2019). Wild bees (Apoidea, Anthophila) represent a highly diverse taxonomic group with significant biological and ecological variety. In France alone, there are approximately 990 species, while Europe hosts over 2,000 species. Globally, the number of wild bee species is estimated to exceed 20,000.

The DYNAFOR Lab (UMR 2021, DYNAmics and ecology of agriFORestry landscapes) has a strong background and skills in landscape ecology. Since 2010, it has been involved in different projects investigating the role of landscape and local factors on wild bees, mostly using pan traps in diversified habitats within agroecosystems.

Sampling started within the framework of the European project BioBio (2009-2012) that aimed at establishing a set of indicators of different instances of biodiversity across a variety of farm types and scales in Europe (13 countries) and two African regions, to assess the status and evolution of farmland biodiversity (Herzog et al. 2013). This study showed that the diversity and abundance of wild bee communities were favoured by the association of a diversity of flowering plants and the presence of little or no anthropogenised herbaceous habitats with areas of naturally bare soil (Lüscher et al. 2014). Amongst the 237 farms studied, the 16 French farms hosted the greatest abundance and species richness of wild bees (Lüscher et al. 2016). Following studies dealt with the wood edge effect on pollinators, including wild bees (Andrieu et al. 2018) with a particular focus on oil seed rape crop showing a decrease in abundance from the wood edge to the field centre (Bailey et al. 2014). At landscape scale, we showed that: 1) the abundance of above-ground nesting bees caught in the border of wheat crop were positively influenced by the presence of steep permanent meadows (Carrié et al. 2018) and that 2) wild bees communities showed trait-based responses of landscape gradients (Carrié et al. 2017b). Pesticide appeared to be detrimental to wild bees caught in field borders, even with a single application (Rivers-Moore et al. 2023). While nitrogen input has a negative effect on wild bee abundance whatever the landscape context, we showed an interaction between the effect of Semi-Natural Habitats (SNH) cover and farming practices intensity assessed at landscape scale (Carrié et al. 2017a). Indeed, we found a positive effect of SNH on wild bee abundance and richness in landscapes exhibiting high and moderate levels of insecticide. We also studied temporal changes in wild bees community structure in forests, with a focus on the role of forest heterogeneity (Andrieu et al., unpublished data). We better characterise the role of SNH types (woods and their edges, hedgerows, grasslands and their relative effects) on wild bees using hunting methods and investigating pollen loaded by wild bees in order to build interaction networks (Rivers-Moore et al. 2020). The knowledge and expertise accumulated on wild bees in south-western France was used to adapt and validate the spatially explicit InVest pollination model. It enables us to provide guidelines on the location of flower patches to optimise pollination services for sunflower crop (Desaegher et al. 2021).

Since 2013, the wild bees caught in the Long Term Socio-Ecological research site ZA PYGAR (Ouin et al. 2021) have been stored in a collection. A fraction of the specimens contained in the collection (534 specimens) and covering 60% of wild bee diversity sampled in ZA-PYGAR, has been used to generate DNA barcoding reference sequences, allowing a cross-validation of the specimen identification (Ollivier et al. In press, Marquisseau et al. In prep.). The aim of this publication is to increase the accessibility and visibility of these datasets to maximise the contribution of these sacrificed specimens to the knowledge and protection of their living conspecifics.

## General description

### Purpose

The database pools 24,329 specimens issued from several research projects for which the sampling method was either pan traps or sweep nets. The database herein presented contains reference identification number for each specimen, taxonomic identification (including specimen identifiers), information on sampling method and location (including year of trapping, catching method, habitat type and coordinates of the trapping location) and the marker used for DNA barcoding when specimens are concerned.

## Project description

### Design description

Sampling was carried out as part of seven research projects (BioBio, Bilisse, Farmland, Sebioref, Muesli, PACSE, Forestbees) which took place in the same geographical area: the "Vallées et Coteaux de Gascogne" site (belonging to the Long Term Socio-Ecological Research site ZA-PYGAR, Ouin et al. (2021)).

## Sampling methods

### Study extent

The LTSER ZA PYGAR site took place in south-western France in the foothills of the Pyrénées Mountains. It is characterised by mixed crops and extensive livestock farming systems. These agricultural features and topographic constraints have designed highly heterogeneous landscapes where wooded elements (hedgerows, scattered trees, small forests) are embedded in a mosaic of annual crops and permanent or temporary meadows (Ouin et al. 2021). According to the scientific question investigated, sample sites were chosen along gradients of landscape heterogeneity (composition and configuration) and production system (conventional to organic farming).

### Sampling description

**Figure 1.**
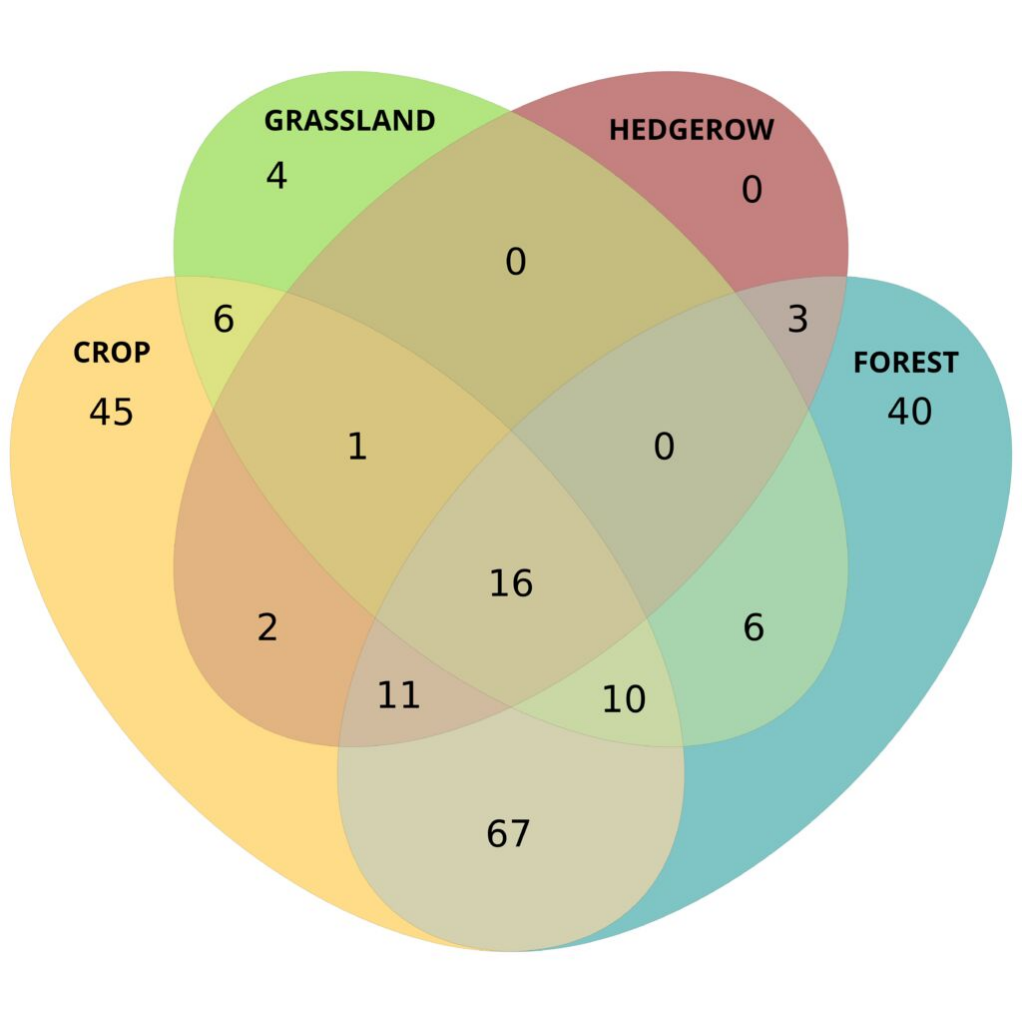
Venn diagram illustrating the number of species sampled in and between each type of habitat: crop (including border and interior), hedgerow, permanent grassland and forest (including forest edge, open canopy and closed canopy). This figure does not show habitats sampled for project BioBbio, for which these data are no longeravailable. Sampling efforts were not the same amongst the different habitat types.

Wild bees were sampled in various habitat types (cultivated and semi-natural, Fig. 1) including field margins of crops and inner crops (12,701 individuals), small forests and their edges (7,856 individuals), hedgerows (119 individuals) and permanent grasslands (294 individuals). The precision of the GPS coordinates given in the dataset has been reduced to preserve the locality of vulnerable species. Depending on the project, two sampling methods were implemented: 1) insect net catching along transects for 3,888 individuals belonging to 183 species (described in Rivers-Moore et al. (2020)) or 2) one to two sets of three to six coloured pan traps per sampling site (blue, yellow and white UV colour paint) (e.g. Rivers-Moore et al. (2023)) for 20,441 individuals belonging to 203 species.

### Quality control

Before 2015, depending on the project and on the people involved, the most obvious and common species were identified by Samantha Bailley, Romain Carrié, Clara Singh and Léa Frontero, all the others being sent to experts (see below), except for the BioBio project for which all specimens were sent to David Genoud.

After 2015, all sampled specimens were sorted out to order and family ranks and pinned, before being sent to national experts of Apoidea apiformes group: Rémi Rudelle, David Genoud, Eric Dufrêne, Alain Pauly and Pierre Rasmont.

For four species complexes (Bombusgr.terrestris, Halictusgr.simplex, *Seladonia* gr. *Smaragdula* and Hylaeusgr.brevicornis), the taxonomy is not stabilised for the female individuals and the current identification tools (dichotomous keys) do not allow for a clear separation of the species within these complexes. Taxonomists agree to use the name of the complex for specimen identification when it could be one of the following species: *Bombusterrestris*, *Bombuslucorum*, *Bombusmagnus* and *Bombuscryptarum* (four species of the Bombusgr.terrestris) , *Halictussimplex*, *Halictuseurygnathus*, *Halictuslangobardicus*, *Halictuscrenicornis*, *Halictustridivisus*, *Halictusadjikenticus*, *Halictuscarinthiacus*, *Halictusgruenwaldti* and *Halictuspyrenaeus* (nine species of the Halictusgr.simplex), *Seladoniasmaragdula*, *Seladoniagemmella* and *Seladoniasubmediterranea* (three species of the Seladoniagr.smaragdula, Pauly et al. (2015)) or one of the 22 species from *Hylaeus* gr. *brevicornis* (Dathe 2022). In the present dataset, the specimens belonging to these groups have been identified either to the species level (when morphological characters allowed) or to its complex species group (for female individuals). Ongoing barcoding initiatives may help to clarify the reliable morphological characteristics for identifying species within these complexes.

Recently, a fraction of the specimens contained in the collection (534 specimens) and covering 60% of wild bee diversity sampled in ZA-PYGAR has been used to generate DNA barcoding reference sequences, allowing a cross-validation of the specimen identification. For each specimen chosen, one front leg was sampled and used for further molecular processes involving DNA extraction, marker amplification and sequencing (articles in prep., Clarke et al. 2014, Hebert et al. 2003, Folmer et al. 1994) . Due to the existence of groups of species particularly recalcitrant to sequencing of the standard CO1 marker, two complementary markers were targeted: CO1 and 16S (Sheffield et al. 2017, Villalta et al. 2021). A total of 110 and 347 specimens were sampled to provide CO1 marker barcodes and 16S marker barcodes, respectively, while 76 specimens were used to generate reference barcodes for both markers. DNA barcoding results are currently being analysed and discussed with experts. In case of incongruence between morphological and molecular identifications, the specimens are subjected to re-observation of morphological characters. This process is in progress.

## Geographic coverage

### Description

**Figure 2.**
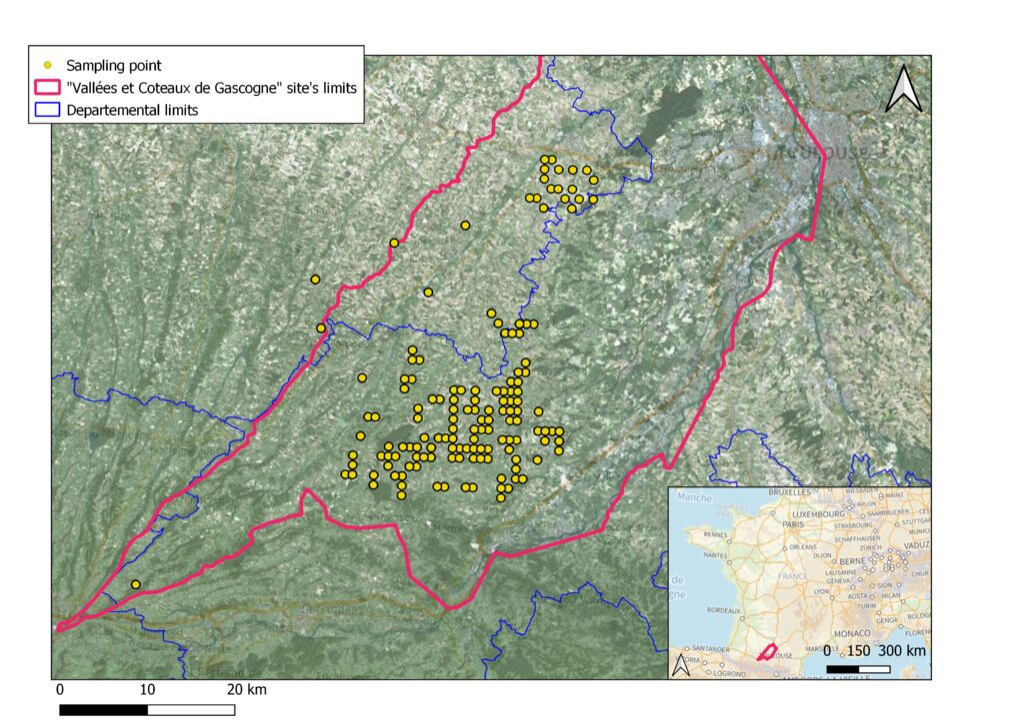
Maps of the geographical distribution of the sampled points in the south of France. Map background: Geoportail and IGN.

The Apoidea specimens come from south-west France, in the LTSER Vallées et Coteaux de Gascogne embedded in the ZA Pygar (Fig. 2). A total of 21,890 specimens were collected in the French Department Haute-Garonne and 2,439 in the Department Gers.

### Coordinates

43.14 and 43.59 Latitude; 0.49 and 1.12 Longitude.

## Taxonomic coverage

### Description

**Figure 3.**
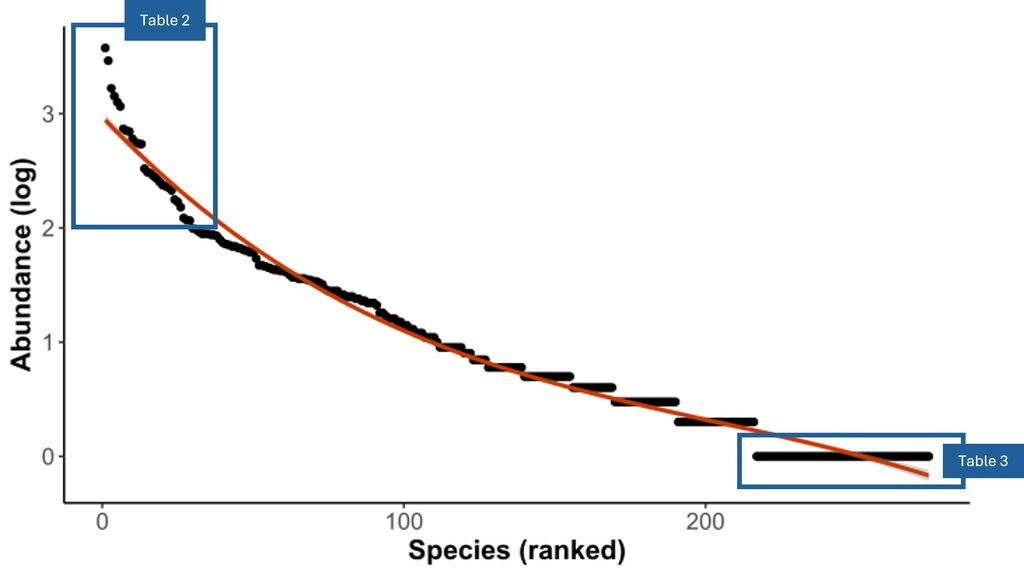
Rank-frequency diagram representing species abundance (log transformed) in the collection. The blue boxes surround the most abundant species (left) and the singletons (right) and refer to the Tables 2 and 3 presenting these species.

The dataset contains at least 270 species of the families Andrenidae (2,958 specimens), Apidae (2,805 specimens), Colletidae (167 specimens), Halictidae (18,007 specimens), Megachilidae (377 specimens) and Melittidae (15 specimens). Thirty-three genera have been identified, the most represented being *Lasioglossum*, *Andrena* and *Halictus* (Table 1). Of the 275 taxa (comprising 270 species and five species complexes), 29 taxa are represented by more than 100 specimens (Fig. 3) and 134 taxa are represented by 1 - 5 individuals, 56 being singletons (Fig. 3). Lists of the common and rare species are provided in Table 2 and Table 3.

## Temporal coverage

**Data range:** 2010-5-26 – 2022-4-12.

### Notes

Sampling is inconsistent over time, depending on the projects being carried out each year and the protocols chosen. The number of sampling events varies from 10 to 452 between 2010 and 2022, with an average of 127 samplings per year over this period.

## Usage licence

### Usage licence

Other

### IP rights notes

CC BY 4.0

## Data resources

### Data package title

Wild bees (Apoidea, Anthophila) of south-west France: more than 10 years of inventories in mosaic landscapes of ZA-PYGAR

### Resource link

https://doi.org/10.15468/dm9eza

### Number of data sets

1

### Data set 1.

#### Data set name

bees_revised.csv

#### Data format

CSV (tab delimited values)

#### Download URL

http://dynids.toulouse.inra.fr:8180/ipt/archive.do?r=baseabeilles

#### Data format version

Darwin Core

#### Description

The dataset includes 24,329 Apoidea specimens from Dynafor’s lab collection (located at INP-ENSAT, Av. de l'Agrobiopole, 31326 Auzeville-Tolosane, FRANCE) (Ollivier et al. 2024).

**Data set 1.** 

Column label	Column description
occurrenceID	Individual identification: combination of year, project name, sampling point name, habitat, trap number and colour, visit number and specimen number in the trap.
eventDate	Sampling date in the format DD-MM-YYYY.
year	Year of sampling.
scientificName	Lowest taxonomic rank possible, usually the species name. If the species is unknown, the genus name is given.
phylum	Phylum.
class	Class.
order	Order.
family	Family.
subfamily	Subfamily.
genus	Genus name.
specificEpithet	Species epithet of the scientificName.
infraspecificEpithet	Infra-species epithet of the scientificName (subspecies).
IdentificationQualifier	Precision or doubts about the identification.
taxonRank	Taxonomic rank of the most specific name in the scientificName, based on TAXREF ranks.
taxonID	Number corresponding to CD_NOM, the unique identifier of the scientific name in TAXREF, the national repository for the taxonomy of the fauna, flora and fungi of France.
sex	Female, Male, Worker or Queen.
identifiedBy	Name of the entomologist(s) who identified the specimen.
dateIdentified	Date of identification, if known, in the format DD-MM-YYYY or YYYY.
decimalLatitude	Geographic latitude (in decimal degrees) of the location, based on the WGS84 coordinate system. The coordinates have been reduced to two digits after the point, to limit precision.
decimalLongitude	Geographic longitude (in decimal degrees) of the location, based on the WGS84 coordinate system. The coordinates have been reduced to two digits after the point, to limit precision.
geodeticDatum	Spatial reference system (SRS) upon which the geographic coordinates given in decimalLatitude and decimalLongitude are based.
country	Country of capture.
stateProvince	French Department name.
CatalogNumber	Box name in the collection.
otherCatalogNumbers	Line and column numbers of the specimen in the box, when known.
recordNumber	Label name of the specimen in the box.
fieldNumber	Name of the sampling point where the specimen was sampled.
habitat	Type of habitat where the specimen was sampled: crop, grassland, hedgerow or forest.
visitNumber	Number of the visit to the same sampling point for a given year.
samplingProtocol	Type of trap used to collect the specimen. PAN TRAP = use of coloured pan traps, NET = use of a sweep net.
trapNumber	Between 1 to 6. Number of the coloured pan trap, allowing us to differentiate traps of the same colour in a given sampling point, if necessary.
trapColour	Pan trap colour (either blue, white or yellow).
individualNumber	Unique number given to the specimen inside the pan trap. In the case of net catching, specimen numbers were given for the whole project.
barcodingMarker	If the individual has been barcoded, the type of marker chosen to produce the barcode (CO1, 16S or both).

## Additional information

Specimen preservation methods: Majority of the specimens are stored, dried and pinned (except a few specimens from the Sebioref project stored and dried in individual tubes). In the BioBio project, some specimens are not stored in the collection because of preservation problems and they have been discarded.

## Figures and Tables

**Table 1.** Number of species and specimens per genus.

**Genus**	**Number of species**	**Number of specimens**
*Amegilla*	1	1
*Andrena*	68	2,951
*Anthidiellum*	1	3
*Anthidium*	2	27
*Anthophora*	4	128
*Bombus*	15	1,486
*Ceratina*	4	98
*Chelostoma*	2	16
*Coelioxys*	1	6
*Colletes*	3	33
*Dasypoda*	1	10
*Eucera*	7	743
*Halictus*	10	2,892
*Heriades*	2	40
*Hoplitis*	3	9
*Hylaeus*	17	134
*Lasioglossum*	39	14,938
*Lithurgus*	2	14
*Megachile*	11	67
*Melecta*	2	2
*Melitta*	3	5
*Nomada*	25	238
*Osmia*	12	175
*Panurgus*	2	7
*Pseudoanthidium*	1	1
*Rhodanthidium*	1	1
*Rophites*	1	2
*Seladonia*	7	133
*Sphecodes*	13	42
*Stelis*	3	11
*Tetralonia*	2	11
*Trachusa*	1	7
*Xylocopa*	3	98

**Table 2.** Taxa (species or species complexes) collected from the ZA PYGAR with more than 100 specimens.

**Taxon**	**Number of specimens**
*Lasioglossummalachurum*	3758
*Lasioglossummarginatum*	2910
*Lasioglossumpallens*	1668
*Halictusgr.simplex*	1426
*Lasioglossumpolitum*	1265
*Halictusscabiosae*	1158
*Lasioglossumcorvinum*	736
*Lasioglossumpauxillum*	709
*Lasioglossumpuncticolle*	698
*Andrenahaemorrhoa*	603
*Andrenaflavipes*	561
*Lasioglossumvillosulum*	546
*Lasioglossumglabriusculum*	542
*Bombusterrestris*	401
*Euceranigrifacies*	330
*Bombuspascuorum*	315
*Lasioglossumalbipes*	301
*Bombuslapidarius*	284
*Lasioglossummorio*	271
*Lasioglossuminterruptum*	253
*Lasioglossumnigripes*	237
*Lasioglossumzonulum*	232
*Andrenaminutula*	213
*Bombusgr.terrestris*	177
*Andrenaangustior*	170
*Andrenanitida*	152
*Euceraclypeata*	122
*Euceranigrescens*	117
*Lasioglossumleucozonium*	116

**Table 3.** List of the singleton taxon collected from the ZA PYGAR (*As these species were identified around ten years ago, they would deserve to be re-observed to check their identification against recently published taxonomic keys, combined with DNA confirmation).

**Taxon**	**Number of specimens**
*Amegillaalbigena*	1
*Andrenabimaculata*	1
Andrenachrysopyga*	1
*Andrenagriseobalteata*	1
Andrenahedikae*	1
Andrenahelvola*	1
*Andrenalabiata*	1
Andrenaleucolippa*	1
*Andrenaminutuloides*	1
*Andrenapallitarsis*	1
*Andrenarhenana*	1
*Andrenarussula*	1
*Andrenasynadelpha*	1
Andrenavetula*	1
*Andrenaviridescens*	1
*Anthophoraplagiata*	1
*Bombuspomorum*	1
*Ceratinadallatorreana*	1
*Chelostomaemarginatum*	1
*Eucerainterrupta*	1
*Halictusfulvipes*	1
*Hoplitisadunca*	1
*Hylaeuscornutus*	1
*Hylaeusrubicola*	1
*Hylaeussinuatus*	1
*Lasioglossumcostulatum*	1
*Lasioglossumgr.tricinctum*	1
*Lasioglossummesosclerum*	1
Lasioglossumsemilucens*	1
*Megachilealbisecta*	1
*Megachileapicalis*	1
*Megachileericetorum*	1
*Megachilemaritima*	1
*Megachilemelanopyga*	1
*Melectaalbifrons*	1
*Melectaitalica*	1
*Melittanigricans*	1
*Melittatricincta*	1
*Nomadahirtipes*	1
*Nomadaflavopicta*	1
*Nomadamelathoracica*	1
*Nomadasuccincta*	1
*Nomadavillosa*	1
*Nomadazonata*	1
*Osmialeaiana*	1
*Panurguscalcaratus*	1
*Pseudoanthidiumscapulare*	1
*Rhodanthidiumseptemdentatum*	1
Seladoniaconfusa*	1
*Sphecodescrassus*	1
*Sphecodesferruginatus*	1
*Sphecodesmarginatus*	1
*Sphecodesmonilicornis*	1
*Sphecodesrubicundus*	1
*Stelisannulata*	1
*Stelispunctulatissima*	1
